# Isolation, identification, and biochemical characterization of five *Lacticaseibacillus* strains from Oggtt: A traditional fermented and dried buttermilk

**DOI:** 10.1002/fsn3.3140

**Published:** 2022-11-12

**Authors:** Ashwak A.‐M. Hassan, Sally S. Sakr, Asmahan A. Ali, Isam A. Mohamed Ahmed, Hany Elkashef

**Affiliations:** ^1^ Dairy Science Department, Faculty of Agriculture Cairo University Giza Egypt; ^2^ Department of Food Science and Human Nutrition College of Agriculture and Veterinary Medicine, Qassim University Buraydah Saudi Arabia; ^3^ Department of Biotechnology and Food Safety National Food Research Centre Khartoum Sudan; ^4^ Department of Food Science and Nutrition College of Food and Agricultural Sciences, King Saud University Riyadh Saudi Arabia

**Keywords:** exopolysaccharides, *Lacticaseibacillus casei*, *Lacticaseibacillus paracasei*, Oggtt

## Abstract

This study investigates the isolation and characterization of the main lactic acid bacteria responsible for fermentation of Oggtt, a dried fermented buttermilk. Five isolates with Gram‐positive staining and negative catalase and oxidase activity were identified using phenotypic and genotypic methods, and their antagonistic, exopolysaccharides and organic acid production, proteolytic activity, and antioxidant capacity were assessed. The isolates are classified as *Lacticaseibacillus paracasei* Ogt_1, *Lacticaseibacillus casei* Ogt_2, *Lacticaseibacillus paracasei* Ogt_3, *Lacticaseibacillus paracasei* Ogt_4, and *Lacticaseibacillus paracasei* Ogt_5. All strains possessed high antagonistic activity against *Proteus vulgaris*, *Staphylococcus aureus,* and *Escherichia coli*. All strains produced high levels of lactic acid (11177.3–15404.9 μg/ml), tartaric acid (2197.8–4058.5 μg/ml), and exopolysaccharides(20.86–239.9 mg/L) and possessed high proteolytic and antioxidant activity at variable manners. Overall, this study indicates the isolation of important *Lacticaseibacillus* strains from Oggtt, which could be used as starter cultures for developing functional foods.

## Graphical abstract

Five Lacticaseibacillus strains were isolated from Oggtt samples. All isolates possessed high antagonistic, antioxidant, and proteolytic activities. All isolates produced sufficient amounts of lactic and tartaric acids and exopolysaccharides. The isolates could be unique starter cultures for developing functional foods.

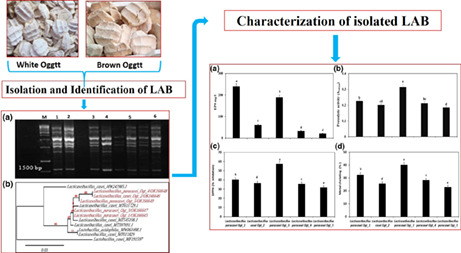

## 1 INTRODUCTION

Lactic acid bacteria (LAB) are a crucial group of bacteria that are able to yield lactic acid through hetero‐ or homofermentative metabolism (Mohamad et al., 2020). Several studies have reported that LAB are able to produce diverse beneficial metabolites including short‐chain fatty acids, essential amino acids, digestive enzymes, water‐soluble vitamins, and antimicrobial agents (Amin et al., 2020; Masuda et al., 2012). The fermentation and enzymatic functions of LAB are considerable for texture, flavor, inhibition of spoilage microorganisms, and extended shelf‐life of fermented food products (Morales et al., 2011).

Various LAB species have isolated from different sources of food such as bovine milk, camel milk, cheese, yogurt, and as well from the gastrointestinal tract of human, bee, and some animals (Kwun et al., 2020). Although the selection of commercial starter LAB for using in food fermentation is a crucial issue, the screening of novel LAB strains from unprecedented sources may be valuable for human health and food industries. The isolation and identification of novel LAB strains aims to reveal the characteristic taxonomy of LAB, obtain promising beneficial and functional probiotic strains particularly from the genus of *Lactobacillus*, and ensure the production of standard quality of fermented food products (Celik et al., 2021). *Lactobacillus* species hold a long history of safe use where they are responsible of the fermentation process together with associated starter cultures or they were added as adjunct cultures (Widyastuti et al., 2021). Most of *Lactobacillus* strains are non‐pathogenic residents in animal and human intestine and their existence is crucial for the protection of gut microflora (Jacobsen et al., 1999).

Oggtt is one of the dried fermented food products with a long shelf‐life due to its a low pH and water activity that is produced traditionally in several Middle East countries including Saudi Arabia, Jordan, Syria, Iraq, and Egypt. It is characterized by high protein and calcium content, low fat percentage, and pleasant organoleptic properties (Hamad et al., 2016). Oggtt is still produced under no modern or standardized protocol. Although Oggtt, Madeer, and Jameed are sun‐dried fermented buttermilk prepared after spontaneously fermented and churning goat, camel, or sheep milk, there is a little difference in processing steps between them (Al‐Abdulkarim et al., 2013). In Saudi Arabia, Oggtt is made by boiling the buttermilk with stirring until it thickens, then thick paste is allowed to cool and shaped before let to the sun for drying (Al‐Abdulkarim et al., 2013; Hamad et al., 2016). In Jordan, Syria, Iraq, and Egypt, the produced buttermilk is let to drain in cheesecloth instead of boiling before shaping and allowing for solar drying (Alu'datt et al., 2016). In both methods, the fermentation is done spontaneously by wild LAB (Hamad et al., 2016). So, these strains can be isolated and identified to be used as pure culture strains for further milk fermentation. However, to date studies on the isolation and characterization of lactic acid bacteria (LAB) from Oggtt are scarce (Al‐Hindi et al., 2015) and consequently the main strains involved in the fermentation of Oggtt remain anonymous. Therefore, the aim of the present work was to isolate and identify novel LAB from Oggtt that produced indigenously in Saudi Arabia.

## 2 MATERIALS AND METHODS

### 2.1 Materials

In Saudi Arabia, there are two types of Oggtt that differed in their color due to variations in manufacturing seasons. The first type is the brown color Oggtt, which is manufactured in summer, while the second type is the creamy color Oggtt which is manufactured in winter. In this study, 40 Oggtt samples (20 brown color Oggtt and 20 creamy color Oggtt) weighing about 200 g each were collected from popular stores in Qassim region and aseptically handled to avoid contamination and transferred in sterilized containers to the laboratory for analysis. All chemicals and microbiological media were of analytical grades and were obtained from Sigma‐Aldrich (Egyptian International Center for Import) and Oxoid Limited Company, respectively.

### 2.2 Isolation and purification of LAB

Ten grams of Oggtt samples were added to 90 ml of sterile 0.1% peptone saline solution supplemented with 0.1% Tween 80 in an aseptic pack and the mixture was homogenized in a stomacher (Lab Blender 400, Seward Medical) for 3 min till homogenous solutions are accomplished to make the initial dilution. Serial dilutions were made for each sample and then 1 ml of the appropriate dilution was inoculated in De Man, Rogosa, and Sharpe (MRS) medium and then incubated at 37°C for 48–72 h. Colonies with distinct morphological differences based on color, shape, size, rough, or smooth surface, were selected and then purified.

### 2.3 Biochemical screening

The presumptive *Lacticaseibacillus* isolates were distinguished using Gram staining, cell morphology, catalase activity, oxidase activity, milk coagulation, and gas production from glucose in MRS broth as previously described by Patil et al. (2010). Under safety conditions, the non‐LAB isolates were discarded. Gram‐positive isolates exhibited negative catalase and oxidase activity was maintained in MRS broth supplemented with 15% glycerol before being stored at −20°C.

### 2.4 Molecular characterization of isolated LAB by 16S rRNA gene sequencing

Bacterial identification by polymerase chain reaction (PCR) and DNA Sequencing of 16S rRNA gene was done. The *Lacticaseibacillus* strains identification was confirmed by 16S rRNA sequence analysis. Initially, chromosomal DNA was extracted from each isolate according to the method mentioned by Ward and Downie (2005). The polymerase chain reaction (PCR) primer used were as follows: 28F 5′AGAGTTTGATCCTGGCTCAG‐3′ (positions 8–28 in *E. coli* numbering) and 1512 R 5′ACGGCTACCTTGTTACGACT‐3′ (positions 1512–1493 in *E. coli* numbering). PCR amplifying protocol was as follows; initial denaturation for 3 min at 95°C for 1 cycle, 40 cycles of 95°C for 30 s, 55°C for 30 s, and 72°C for 1 min, and a final extension cycle of 72°C for 10 min.

PCR amplified DNA segments were separated by electrophoresis on 0.8% agarose gel and visualized using ethidium bromide. Product size was determined by comparison with a DNA 1 KB ladder. Products were purified using QIA quick PCR Purification Kit according to the manufacturer's instructions. For verifying the purification, the purified products were run on 0.8% agarose gel before the Sequencing of the PCR fragments which was conducted by Cairo University Research Park (a commercial service provider). Databases were screened for similarities by using BLAST program (NCBI). A Phylogenetic tree was constructed based on 16S rRNA gene sequences of the five strains using the ‘Phylogeny.fr’ online tool (Dereeper et al., 2008). The tree showed the relationship between the isolates and other closely related sequences of NCBI GenBank reference taxa.

### 2.5 Organic acid profile of isolated LAB

The organic acids in the cell‐free supernatant (CFS) were analyzed according to the method described by Parlindungan et al. (2021). An amount of 20 μl of the sample was injected into an Agilent 1200 high‐performance liquid chromatography (HPLC) system with a Refractive Index Detector and a REFEX 8 μm 8% H Organic Acid Rezex@ column (Phenomenex). The elution fluid was H_2_SO_4_ (5 mmol/L) at a flow rate of 0.6 ml/min with the temperature of the column retained at 65°C. MRS broth was also analyzed as a control. Authentic standards of organic acids were run under the same conditions, and the retention time of peaks of the samples was compared with those of organic acid standards and quantified by calculating area under the peaks.

### 2.6 Exopolysaccharides (EPS) of isolated LAB

EPS were determined following the method described by Welman and Maddox (2003). Briefly, LAB isolates were grown in MRS broth at 37°C for 16–18 h to attain absorbance (A600) of 0.9, and then inoculated at 1% (v/v) in modified MRS broth, in which, glucose (20 g/L) was replaced with sucrose, and fermentation was allowed to proceed for 72 h at 37°C. Then, modified MRS agar was inoculated with 1% (v/v) fresh culture using pour plate technique and the stickiness of colonies grown in modified media agars was determined by the inoculating loop method. EPS were assayed in the supernatant. Culture supernatant obtained after centrifugation (11,000 ***g*** for 10 min) was added to two volumes of cold ethanol, and allowed to stand for 12 h at 4°C until precipitation take place. The precipitate was separated by centrifugation (2500 ***g***, 20 min) and suspended in distilled water, followed by the addition of 2 volumes of cold ethanol. Samples were centrifuged at 2500 ***g*** and the EPS pellets were dried at 60°C and weighed.

### 2.7 Proteolytic activity of isolated LAB

The Proteolytic activity of the identified *Lacticaseibacillus* strains was evaluated using the method of Shori et al. (2013). Briefly, 10 g of skimmed milk was fermented by identified *Lacticaseibacillus* strains and then 40 ml distilled water was added and mixed in a screw‐cup tubes before homogenization. The homogenates were then incubated for 1 h at 40°C. The obtained homogenates were centrifuged at 10000 ***g*** (Hermle Labortechnik GmbH, Wehingen, Germany centrifuge with SER. #220.72, Type 09/144 rotor) at room temperature (22–25°C) for 30 min and the water‐soluble extract (WSE) was withdrawn. The proteolytic activity of WSE was estimated by O‐phthaldialdehyde (OPA) method using UV/Visible spectrophotometer (Jenway® Genova Life Science, Bibby Scientific Ltd.) at 340 nm.

### 2.8 Antioxidant activity of isolated LAB

The antioxidant activity of milk fermented by different identified *Lacticaseibacillus* isolates was estimated using DPPH radical scavenging activity and metal ions chelating activity according to the methods described by Jemil et al. (2016).

### 2.9 Antagonistic activity of isolated LAB

The antagonistic activity of the pre‐identified *Lacticaseibacillus* strains against some pathogenic microbes was also evaluated. The pathogenic bacteria used were: *Escherichia coli* ATCC 25922, *Proteus vulgaris* ATCC13315, *Staphylococcus aureus* ATCC25923, and *Bacillus subtilis* NRRL B‐543, as well as 2 fungal strains (*Aspergillus fumigatus* RCMB 002008 and *Candida albicans* ATCC25923). The antagonistic activity was evaluated for separate strains using the agar well diffusion. Briefly, 100 μl of the pathogenic bacteria suspension (10^8^ CFU/ml) was spread over the entire agar surface of Mueller Hinton (MH) and a 6‐mm diameter well was punched aseptically onto the MH agar. After that, 100 μl of MRS broth containing an overnight cultured LAB isolates was seeded into the well and the plate was incubated at 37°C for 24–48 h. Inhibition zones diameter indicating antagonistic activity of LAB were measured with a ruler held against the back of the Petri plate and the measurements were calculated in millimeter (mm) as follow:

$$\begin{array}{c} \text{Inhibition Zone}\left(\mathrm{mm}\right)=\\ \text{Diameter of growth inhbited zone}-\text{Diameter of the well} \end{array}$$

The obtained results were taken as mean ± SD and calculated from three independent experiments. As previously described, the inhibition zone was scored as strong inhibition, moderate inhibition, and weak inhibition if the diameter was >6 mm, between 3 and 6 mm and <3 mm, respectively (Pan et al., 2009).

### 2.10 Statistical analysis

Results of triplicate samples were collected and statistically analyzed using analysis of variance (anova) by MSTAT‐C software (Michigan State University). In all data, means are shown as means ± SD of three replicates. Differences between means are deemed statistically significant at *p* ≤ .05. Principal component analysis (PCA) and hierarchical cluster analysis (HCA) were carried out using a MULTBIPLOT software as stated in the instruction manual (Vicente‐Villardón, 2010).

## 3 RESULTS AND DISCUSSION

### 3.1 Isolation and identification of LAB

In this study, 25 isolates of LAB were obtained from Oggtt samples. Of them, five isolates (Ogt_1, Ogt_2, Ogt_3, Ogt_4, and Ogt_5) with different morphological characteristics were selected for further analysis. Upon incubation of the isolates in MRS Microaerophiles agar media for 24 h, creamy, opaque, and circular colonies were observed for the isolates Ogt_1, Ogt_3, and Ogt_4, whereas Ogt_2 exhibited milky white, rounded, and smooth colony shape and Ogt_5 colonies appeared as grayish‐white in color with smooth edges (Table 1). Coagulation test was carried out by inoculating the isolates into sterile skim milk and observation of coagulation was recorded at intervals of 6, 9, and 12 h. All isolates showed good coagulation properties without causing textural defects such as gas bubbles. The isolates of Ogt_1, Ogt_2, Ogt_3, and Ogt_4coagulated milk after 9 h, whereas Ogt_5 coagulated it after 12 h. Moreover, changes in milk pH were recorded after 24 h of inoculation for all isolates. The pH of the blank was 6.48, where the pH values of Ogt_1, Ogt_2, Ogt_3, Ogt_4, and Ogt_5 were 4.61, 4.60, 4.64, 4.64, and 4.76, respectively. None of the five isolates produced carbon dioxide from glucose and these results confirmed the homofermentative characteristics of the isolated strains (Galli et al., 2022). Homofermentative LABs are of high importance for the production of high‐quality lactic acid for food and pharmaceutical applications in addition to their probiotic properties (Galli et al., 2022; Moon et al., 2012).

**TABLE 1:** Phenotypic characterization of lactic acid bacteria isolated from Oggtt

Bacterial strains	Ogt_1	Ogt_2	Ogt_3	Ogt_4	Ogt_5
Gram staining	+	+	+	+	+
Catalase test	−	−	−	−	−
Oxidase test	−	−	−	−	−
Colony morphology	Creamy, circular colonies, smooth‐edged	Milky white, big colonies, rounded, smooth	Creamy, circular colonies, smooth‐edged	Creamy, circular colonies, convex elevation smooth‐edged	Grayish‐white, pinpoint, colony, convex elevation, smooth‐edged
Cell morphology	Rods non‐motile, non‐sporulated form chains	Slender rods non‐motile, non‐sporulated, form chains	Rods non‐motile, non‐sporulated form chains	Rods non‐motile, non‐sporulated form chains	Rods non‐motile, non‐sporulated form chains

**FIGURE 1:**
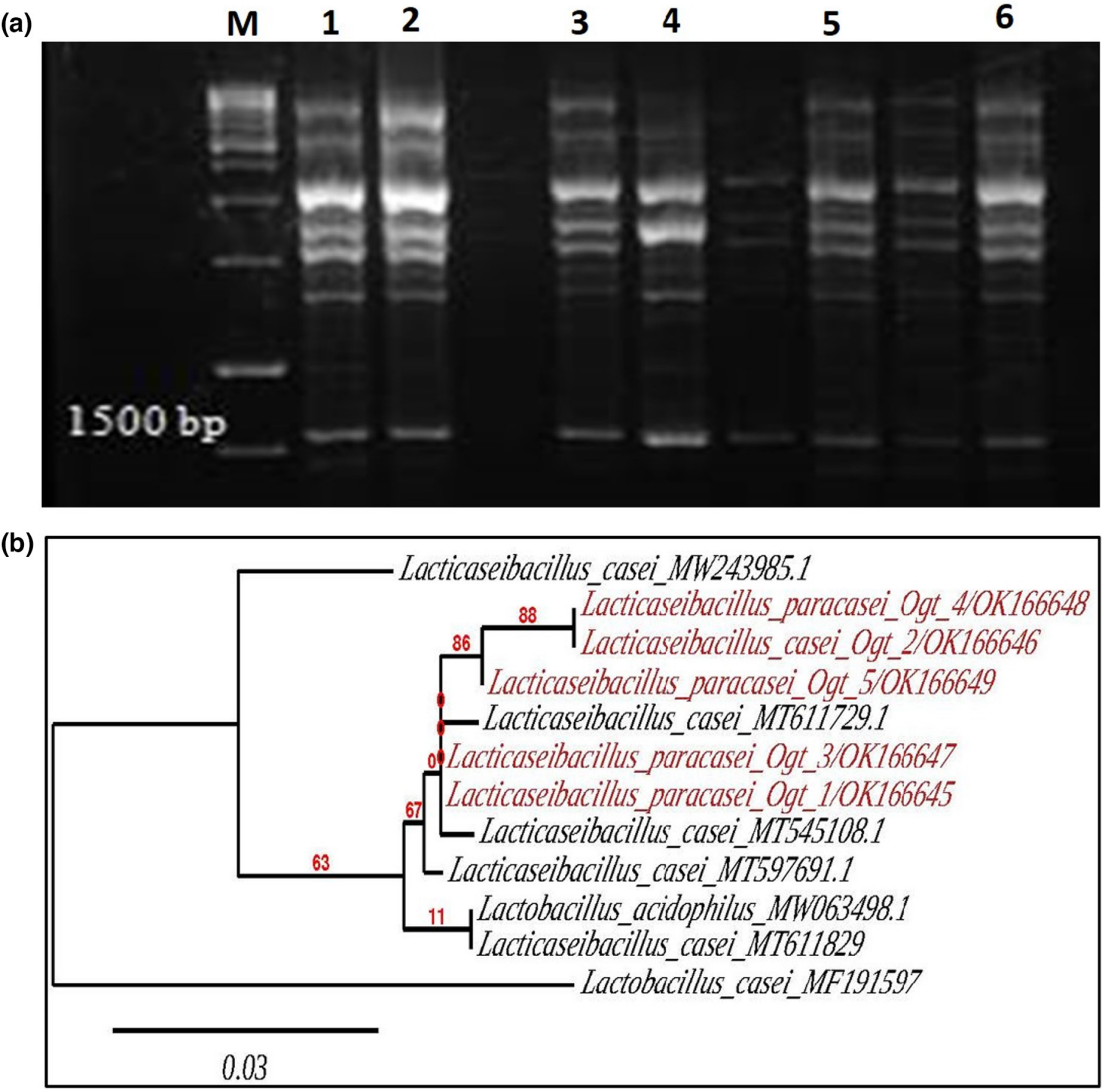
Identification of the main LAB responsible for fermentation of Oggtt using 16S rRNA gene. (a) The detected PCR products on gel electrophoresis for amplified segments of 16S rRNA gene. Lane M: 1 Kbp ladder, Lane 1–5, amplicons of 16S rRNA gene of the isolates Ogt_1, Ogt_2, Ogt_3, Ogt_4, and Ogt_5, respectively, and Lane 6: negative control. (b) The phylogenetic analysis of *Lacticaseibacillus* strains isolated from Oggtt samples. Phylogenetic tree based on a distance matrix analysis of 16S rRNA gene. Closely related type and reference strains are shown in parentheses together with accession numbers from Gene Bank.

In order to identify the five chosen isolates, partial 16S rRNA sequence analysis was conducted using two universal primers (Weisburg et al., 1991), following phenotypic characterization. Each of the chosen isolates generated a PCR product at 1500 bp (Figure 1a). These PCR products were then purified using QIA quick PCR Purification Kit and DNA sequencing of the purified products was done. The GenBank database (NCBI) was then used to search for 16S rRNA sequence similarities and the sequence analysis (BLAST) of all isolates clearly indicated the identity of the organisms. The isolates of Ogt_1, Ogt_3, Ogt_4, and Ogt_5 were molecularly identified as members of *Lacticaseibacillus paracasei*, while isolate of Ogt_2 was identified as *Lacticaseibacillus casei* (Table 2). Strains were submitted to NCBI and had a newly accession number which are OK166645.1 (*Lacticaseibacillus paracasei* Ogt_1), OK166646.1 (*Lacticaseibacillus casei* Ogt_2), OK166647.1 (*Lacticaseibacillus paracasei* Ogt_3), OK166648.1 (*Lacticaseibacillus paracasei* Ogt_4), and OK166649.1 (*Lacticaseibacillus paracasei* Ogt_5).

**TABLE 2:** Molecular identification of the selected bacterial isolates

Isolate	Accession number	Sequence lengths (bp)	16S rRNA	Max. Ident. (%)
Ogt_1	OK166645.1	937	*Lacticaseibacillus paracasei*	99.79
Ogt_2	OK166646.1	980	*Lacticaseibacillus casei*	99.49
Ogt_3	OK166647.1	913	*Lacticaseibacillus paracasei*	99.89
Ogt_4	OK166648.1	869	*Lacticaseibacillus paracasei*	100
Ogt_5	OK166649.1	968	*Lacticaseibacillus paracasei*	99.69

Further evidence of the closeness of the isolates to *Lacticaseibacillus* was obtained by reconstructing a phylogenetic tree (Figure 1b). The robustness, reliability, and ease of construction of the Phylogeny employment in the phylogenetic trees made it a valuable tool in tree reconstruction (Dereeper et al., 2008). One out of groups, namely *Lacticaseibacillus casei* and *Lacticaseibacillus paracasei* were included in the phylogenetic tree. Furthermore, the results coincided with those obtained by gene sequencing, which showed that the 16s rRNA sequences of Ogt_1 to Ogt_5 were highly similar to those of *Lacticaseibacillus casei* and *Lacticaseibacillus paracasei*. Therefore, the isolates are positively identified as *Lacticaseibacillus paracasei* (Ogt_1, Ogt_3, Ogt_4, and Ogt‐5) and *L*. *casei* (Ogt_2). Previously, 50 LAB strains isolated and identified from Oggtt samples using phenotyping and biotyping methods, of which, 30%, 22%, 16%, 14%, 12%, and 6% were identified as *Lactobacillus casei*, *Lactobacillus acidephilus*, *Enterococcus faecium*, *Lactobacillus plantarum*, *Lactobacillus lactis*, and *Lactobacillus fermentum*, respectively (Al‐Hindi et al., 2015). The variation in the LAB composition between these studies could be attributed to the difference in the milk type used for preparation of Oggtt, processing method and condition of Oggtt, season and storage conditions. Homofermentative strains of *Lacticaseibacillus casei* and *Lacticaseibacillus paracasei* are known for their potential applications in the production of high‐purity lactic acid and functional probiotic foods (Galli et al., 2022; Moon et al., 2012). Hence, the identified strains could be of potential application in the production of functional foods.

### 3.2 Organic acids produced by the isolated strains

Identification of organic acids as one of the important components produced by lactic acid bacteria strains was conducted to understand the mechanisms of action behind antimicrobial and therapeutic characteristics of these bacteria and their ability for producing specific organic acids. Thus, the production of some organic acids such as oxalic, tartaric, succinic, lactic, acetic, butyric, and propionic was investigated (Table 3, Figure S1). Results showed that lactic, tartaric, oxalic, and succinic were present in large amounts in all the fermentation media compared to the other acetic, butyric, malic, and propionic acids. Among the isolated strains, *Lacticaseibacillus paracasei* Ogt_5 produced the highest concentration of lactic, tartaric, and oxalic acids, whereas the least values of these acids were produced by *Lacticaseibacillus paracasei* Ogt_4. In addition, *Lacticaseibacillus casei* Ogt_2 produced the highest levels of succinic and propionic acids, and also share the highest level of acetic acid with *Lacticaseibacillus paracasei* Ogt_3. Interestingly, malic and butyric acids were only produced by *Lacticaseibacillus paracasei* Ogt_3. Variations in the content and composition of organic acids among the isolated strains could be attributed to the variations in the genetic makeup and metabolic pathways among these strains. Similarly, previous reports indicated that lactic acid as the main organic acid that produced by homofermentative LAB (da Costa et al., 2018; Zalán et al., 2010). In addition, LAB produced numerous organic acids such as lactic, acetic, formic, citric, succinic, and glutamic acids when grown on MRS media (Zalán et al., 2010). The findings of this study indicated that the presence of LAB in food matrix plays a crucial role in controlling spoilage and/or pathogenic microorganisms through the production of organic acids. However, it is important to note that organic acids may not be the sole contributing factors since LAB may also produce a variety of antimicrobial compounds including bacteriocins, hydrogen peroxide, carboxylic acid, diacetyl, and reuterin (Stoyanova et al., 2012).

**TABLE 3:** Concentration of organic acids (μg/ml) in the cell free supernatant produced by *Lacticaseibacillus* isolates

Organic acid (μg/ml)	*Lacticaseibacillus paracasei* Ogt_1	*Lacticaseibacillus casei* Ogt_2	*Lacticaseibacillus paracasei* Ogt_3	*Lacticaseibacillus paracasei* Ogt_4	*Lactobacillus paracasei* Ogt_5
Oxalic	600.7^d^	608.4^c^	670.4^b^	480.8^e^	817.3^a^
Tartaric	3260.3^b^	2545.5^d^	3138.7^c^	2197.8^e^	4058.5^a^
Succinic	241.3^c^	572.2^a^	191.0^d^	375.5^b^	184.0^e^
Lactic	13057.2^c^	14762.3^b^	12801.7^d^	11177.3^e^	15404.9^a^
Acetic	3.3^b^	4.0^a^	4.1^a^	2.1^c^	1.9^c^
Malic	ND	ND	589.8	ND	ND
Butyric	ND	ND	0.5	ND	ND
Propionic	ND	1.2^a^	0.2^c^	0.9^b^	ND

*Note:* ^a–e^Means with different superscript letters within each row are significantly different at *p* ≤ .05.

Abbreviation: ND, not detected.

### 3.3 Exopolysaccharides produced by identified strains

**FIGURE 2:**
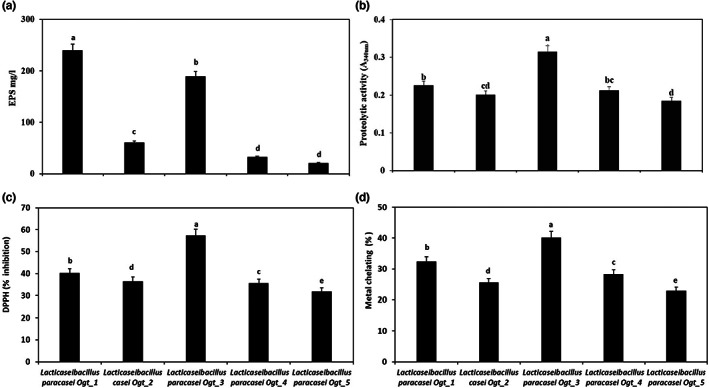
Characterization of LAB strains isolated from Oggtt samples. Production of exopolysaccharides (a), proteolytic activity (b), DPPH radical scavenging activity (c), and metal chelating activity (d) of milk fermented with the isolated LAB strains. Different lowercase letters (a–e) on the bars indicate significant differences at *p* ≤ .05.

Exopolysaccharides (EPS) are multifunctional compounds that have interesting applications in both pharmaceutical and food industries. The averages of EPS were 239.9, 60.57, 189.3, 32.47, and 20.86 mg/L for *Lacticaseibacillus paracasei* Ogt_1, *Lacticaseibacillus casei* Ogt_2, *Lacticaseibacillus paracasei*Ogt_3, *Lacticaseibacillus paracasei* Ogt_4, and *Lacticaseibacillus paracasei* Ogt_5 strains, respectively (Figure 2a). The highest levels of EPS were produced by *Lacticaseibacillus paracasei* Ogt_1, whereas the lowest values were produced by *Lacticaseibacillus paracasei* Ogt_5 indicating great differences in the EPS production among the isolated strains (*p* ≤ .05). The variation in the EPS among the isolated strains is likely due to the differences in the genetic makeup, EPS production pathways, and EPS types. Interestingly, the values of EPS in the isolated strains are within the range 10–400 mg/L produced by LAB under non‐optimized conditions, which could be increased by twofold under optimal growth conditions (Korcz & Varga, 2021). EPS produced by LAB can affect the stability and sensory quality of fermented milk products as they act as texturizers and stabilizers, increasing the viscosity and mouthfeel of products (Galli et al., 2022; Korcz & Varga, 2021). In addition, EPS have high bioactive properties and possess antioxidants, antimicrobial, antiviral, anticoagulant, immunomodulating, and cholesterol‐lowering effects (Zhou et al., 2019). Thus, the findings of this study indicate the potential application of the isolated strains in the development of functional foods and pharmaceutical products.

### 3.4 Proteolytic activity of the identified strains

The proteolytic activity of identified *Lacticaseibacillus* strains cultivated in sterilized skim milk was determined using the OPA method and the results are illustrated in Figure 2b. The proteolytic activity altered among the different *Lacticaseibacillus* strains. *Lacticaseibacillus paracasei* Ogt_3 had significantly highest activity, while *Lacticaseibacillus paracasei* Ogt_5 showed significantly lowest proteolytic activity (*p* ≤ .05). Variation in proteolytic ability could be due to the variation of the proteolytic system among the identified *Lacticaseibacillus* strains. Kieliszek et al. (2021) mentioned that thermophilic *Lactobacillus* species have powerful proteolytic activity compared to *Streptococcus* and rods species, and each species possess strains with highly different ability in this regard. In addition, García‐Cano et al. (2019) isolated several *L. casei* and *L. paracasei* with high proteolytic and lipolytic activities from dairy products. The variation of proteolysis among LAB enables their widespread utilize in several industries. In the dairy industry, the proteolytic activity plays a crucial role in achieving the rheological, sensory and health potential effects of the end products. Thus, the isolated strains could be used as a source of proteolytic enzymes for food and pharmaceutical applications.

### 3.5 Antioxidant activity of the identified strains

The antioxidant activity of *Lacticaseibacillus* strains was assessed by measuring the DPPH radical scavenging (Figure 2c) and metal chelating (Figure 2d) activities to determine the effect of identified strains on the functional properties of fermented milk. The results showed that all the identified isolates exhibited antioxidant ability as assessed by both methods. The highest antioxidant activity was observed in milk fermented with *Lacticaseibacillus paracasei* Ogt_3, followed by *Lacticaseibacillus paracasei* Ogt_1, while the least activity was noted in milk fermented with *Lacticaseibacillus paracasei* Ogt_5. These results suggested that *Lacticaseibacillus paracasei* Ogt_3 and *Lacticaseibacillus paracasei* Ogt_1 generated peptides with the highest radical scavenging and metal chelating activities than those of other identified isolates. In addition, the antioxidant activity of these strains could be linked to the EPS that produced in high quantities in these strains compared to other isolates. Hydrolysis of milk protein by proteolytic enzymes of *Lacticaseibacillus* strains produced antioxidant peptides during milk fermentation. Furthermore, antioxidant peptides can perform as electron donors and might interact with free radicals to transform them into more stable. In addition, fermented milk may include peptides or amino acids that can frustrate lipid oxidation by the capacity of non‐phosphorylated groups to chelate metal ions or by free radical scavenging. The peptides containing basic or acidic amino acids may have a crucial role in the chelating of Cu^2+^ and Fe^2+^ (Farvin et al., 2010). Chooruk et al. (2017) found that all tested *Lactobacillus* strains showed antioxidant activity and *L. paracasei* had high DPPH radical scavenging activity. Also, Chen et al. (2015) screened 25 strains of *Lactobacillus* strains to ability of antioxidant, and they reported that *L. plantarum* L60, *L. casei* L49 and L61, and *L. acidophilus* La5 exhibited high scavenging rate of DPPH radical. Overall, high antioxidant activity of the isolated strains indicates the potentials of these strains in food and pharmaceutical applications.

### 3.6 Antagonistic activity of identified Lactobacillus strains

The identified *Lacticaseibacillus* strains were further evaluated for their antagonistic activity against the following pathogenic microorganisms: *Aspergillus fumigatus* RCMB 002008, *Candida albicans* ATCC 10231, *Staphylococcus aureus* ATCC 25923, *Bacillus subtills* NRRL B‐543, *Escherichia coli* ATCC 25922, and *Proteus vulgaris* ATCC 13315. The pathogens strains (Gram‐positive and Gram‐negative bacteria and fungi) were purposefully selected to represent the target area of the study, which included the microbial inhibition of entero pathogens, food‐borne pathogens, and spoilage micro‐organisms (contaminants). Results of antimicrobial activity of identified *Lacticaseibacillus* strains against six pathogenic microbes are shown in Table 4. All *Lacticaseibacillus* strains isolated from the Oggtt samples showed excellent antagonistic activity against *Proteus vulgaris* ATCC 13315, *Staphylococcus aureus* ATCC 25923, and *Escherichia coli* ATCC 25922, although the degree of antagonism varied among the strains. Interestingly, strains of Ogt_1, Ogt_2, Ogt_3, Ogt_4, and Ogt_5 showed a strong antagonistic activity against *Proteus vulgaris* ATCC 13315 since the diameters of the observed inhibition zones were largely higher than 6 mm. Strains of *Lacticaseibacillus casei* Ogt_2 and *Lacticaseibacillus paracasei* Ogt_4 showed the highest antagonistic activity against *Proteus vulgaris* ATCC 13315 with an average diameter of inhibition of 24.0 mm. All strains, except *Lacticaseibacillus paracasei* Ogt_5, showed no inhibitory effect against *Bacillus subtilis* NRRL B‐543. The inhibition of these pathogenic strains is likely due to the production of metabolites such organic acids and bacteriocins by the isolated strains. No antifungal activity was detected against all examined fungi used in this study. In agreement with our findings, previous studies indicated that *L*. *casei* and *L*. *paracasei* possessed antagonistic activity against various pathogenic bacterial strains (Belguesmia et al., 2020; da Costa et al., 2018; Mechai et al., 2020). The antagonistic activity of these strains was attributed to the production of organic acids (da Costa et al., 2018) and bacteriocins (Belguesmia et al., 2020; Mechai et al., 2020). The antagonistic activity of the isolated strains could indicate the potential applications of these strains as biopreservation tools in fresh food products.

**TABLE 4:** Antimicrobial activity of identified *Lacticaseibacillus* isolates

Pathogenic microbes	Inhibition zone (mm) (means ± SD)				
	Ogt_1	Ogt_2	Ogt_3	Ogt_4	Ogt_5
Fungi					
*Aspergillus fumigatus* RCMB 002008	–	–	–	–	–
*Candida albicans* ATCC 10231	–	–	–	–	–
Gram‐positive bacteria					
*Staphylococcus aureus* ATCC 25923	15.8 ± 0.529^a^	13.7 ± 0.513^c^	15.9 ± 0.95^a^	14.2 ± 1.04^b^	15.9 ± 1.12^a^
*Bacillus subtilis* NRRL B‐543	–	–	–	–	11.6 ± 0.50^a^
Gram‐negative bacteria					
*Escherichia coli* ATCC 25922	19.6 ± 0.57^a^	18.5 ± 0.57^b^	18.2 ± 1.41^bc^	17.4 ± 0.70^c^	18.1 ± 0.57^bc^
*Proteus vulgaris* ATCC 13315	22.2 ± 1.52^c^	24.0 ± 1.73^a^	23.1 ± 1.57^b^	24.0 ± 1.15^a^	23.0 ± 0.0^b^

*Note:* ^a–c^Means with different superscript letters within each row are significantly different at *p* ≤ .05.

### 3.7 Principal component analysis (PCA) and hierarchical clustering analysis (HCA)

**FIGURE 3:**
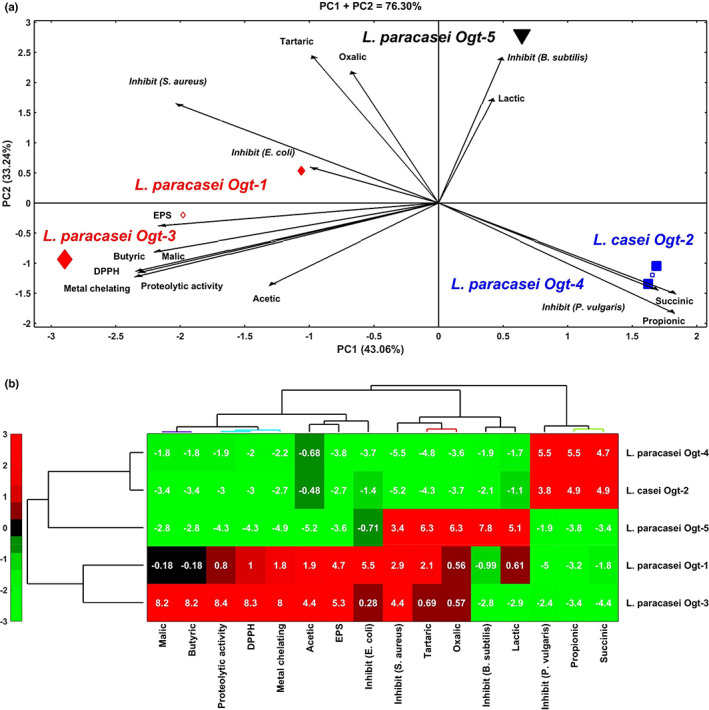
Principle component analysis (PCA) HJ‐biplot (a) and hierarchical cluster analysis (HCA) heatmap (b) of five *Lacticaseibacillus* strains isolated from Oggtt samples.

To deeply assess the overall interrelationship between the isolated strains based on their biochemical, antimicrobial, and antioxidant properties, PCA and HCA were performed the results were presented in Figure 3. In the HJ‐biplot (Figure 3a) the similarity between the isolated strains is indicated by the distance between them, in which, short distance indicates similarity, whereas, long distance indicates dissimilarity (Yan & Fregeau‐Reid, 2008). In this regard, very short distance between *Lacticaseibacillus casei* Ogt_2 and *Lacticaseibacillus paracasei* Ogt_4 indicate high phenotypic similarity between them. In addition, moderate distance between *Lacticaseibacillus paracasei* Ogt_1 and *Lacticaseibacillus paracasei* Ogt_3 indicates moderate phenotypic similarity between them, whereas long distance between *Lacticaseibacillus paracasei* Ogt_5 and all other isolates indicates high phenotypic dissimilarity of this strain from other strains. Consequently, three clusters of isolates were formed based on the similarity and dissimilarity in the phenotypic characteristics between them. The first cluster (black triangle symbol) is for the isolate *L*. *paracasei* Ogt_5 that is characterized by higher amounts of lactic, tartaric, and oxalic acids and possessed higher antimicrobial activity (inhibition zone) against *Bacillus subtilis* compared to all other isolates. The second cluster (blue square symbol) is composed of the isolates *L*. *casei* Ogt_2 and *L*. *paracasei* Ogt_4 those characterized by higher antimicrobial activity against *Proteus vulgaris* and produced more succinic and propionic acids than other isolates. The last cluster (red diamond symbol) is composed of the isolates *L*. *paracasei* Ogt_1 and *L*. *paracasei* Ogt_3 which were characterized by higher antimicrobial activity against *Escherichia coli* and *Staphylococcus aureus*, proteolytic activity, and antioxidant activity (DPPH antiradical activity and metal chelating activity) and produced higher amounts of exopolysaccharides (EPS), malic, butyric and acetic acid. Within this group, *L*. *paracasei* Ogt_3 outscore *L*. *paracasei* Ogt_1 for almost all traits, with few exceptions. Hierarchical cluster analysis (HCA) was also performed to profoundly understand the interrelationship among the isolated strains based on the phenotypic characteristics and HCA heatmap is shown in Figure 3b. The color indicates the similarity between the isolates, in which, red color indicates similarity (high linkage), whereas green color indicates dissimilarity (low linkage). It is clear that *L*. *casei* Ogt_2 and *L*. *paracasei* Ogt_4 share high similarity due to their almost similar inhibition activity against *P*. *vulgaris* and production of more succinic and propionic acids than others do. *L*. *paracasei* Ogt_3 and *L*. *paracasei* Ogt_1 also showed some phenotypic similarly for most attributes with the highest values being observed in *L*. *paracasei* Ogt_3 with some exceptions. *L*. *paracasei* Ogt_5 is more dissimilar than others despite of some low linkage with *L*. *paracasei* Ogt_1 for tartaric acid and inhibition of *S*. *aureus* and in the later, with *L*. *paracasei* Ogt_3, however, *L*. *paracasei* Ogt_3 outscore the others isolates in inhibition of *S*. *aureus*. Overall, the isolated strains exhibited different phenotypic characteristics and application potentials.

*Lacticaseibacillus paracasei* Ogt_3 outscore all other isolates due to its greater antioxidant, and proteolytic activities and production of good amounts of EPS, and butyric, malic, and acetic acids.

## 4 CONCLUSIONS

In conclusion, no doubt that lactic acid bacteria play a crucial role in food industry and human nutrition. Therefore, the isolation and identification of new LAB strains from novel sources are considered significant to be suitable in manufacturing of fermented food products as adjunct starter cultures. The present study provides proof for opportunities to avail Oggtt product, dried fermented milk, as comparatively unexplored sources for the isolation and identification of novel LAB strains for their possibility to utilize as adjunct cultures. We successfully isolated, identified, and characterized five *Lacticaseibacillus* strains, and illustrated their antagonistic, exopolysaccharides production, proteolytic and antioxidant abilities. According to 16S rRNA gene sequences, potential LAB was identified as *Lacticaseibacillus paracasei* Ogt_1, *Lacticaseibacillus casei* Ogt_2, *Lacticaseibacillus paracasei* Ogt_3, *Lacticaseibacillus paracasei* Ogt_4, and *Lacticaseibacillus paracasei* Ogt_5. All isolated *Lactobacillus* strains exhibited excellent antimicrobial activity against *Proteus vulgaris*, *Staphylococcus aureus*, and *E. coli* with different degrees. *Lacticaseibacillus paracasei* Ogt_1 was the highest producer of exopolysaccharides, while *Lacticaseibacillus paracasei* Ogt_3 possess good proteolytic and antioxidant activities. The present work proves that Oggtt product is a rich source of LAB that might have a range of various benefits and applications in fermented food industry. Also, this study provides vigorous support for further in vitro and in vivo research to investigate the beneficial effects of identified strains and their utilization in fermented food products.

## CONFLICT OF INTEREST

The authors declare that they do not have any conflict of interest.

## ETHICS STATEMENT

These data were generated from a fermented food, and therefore no ethics approval was needed.

## Supporting information


Figure S1

## Data Availability

The data used to support the findings of this study are available from the corresponding author upon request.
