# Supplementary material for: Isolation, identification, and biochemical characterization of five Lacticaseibacillus strains from Oggtt: A traditional fermented and dried buttermilk
Source: Food Sci Nutr. 2022 Nov 12;11(2):1040–50. doi: 10.1002/fsn3.3140 (PMC9922129; doi:10.1002/fsn3.3140)
Supplement: Supplementary file 1 — Figure S1 [file FSN3-11-1040-s001.docx]

Supplementary figure


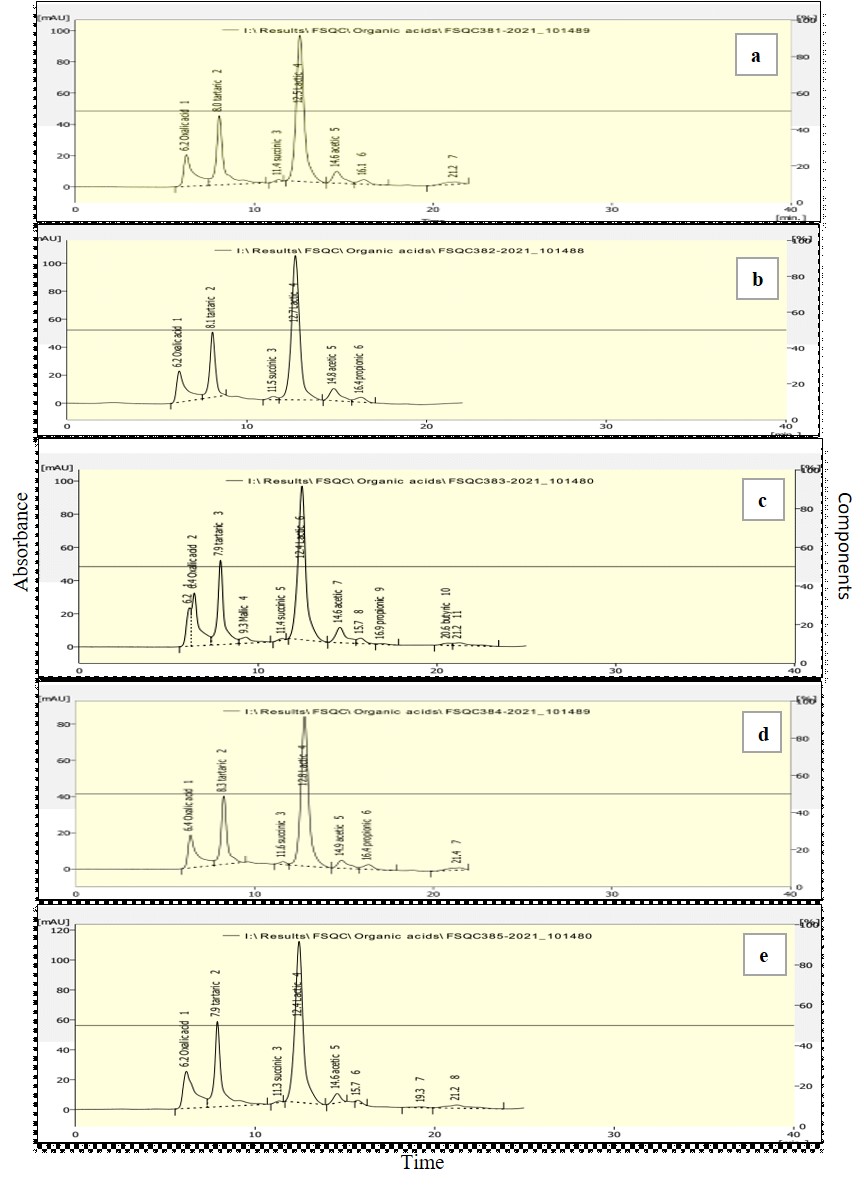


**Fig. S1.** HPLC chromatograms of organic acids from the fermenting medium by strains *Lacticaseibacillus paracasei* Ogt_1 (a), *Lacticaseibacillus casei* Ogt_2 (b), *Lacticaseibacillus paracasei* Ogt_3 (c), *Lacticaseibacillus paracasei* Ogt_4 (d), and *Lacticaseibacillus paracasei* Ogt_5 (e).
